# Anti-Warburg Mechanism of Ginsenoside F2 in Human Cervical Cancer Cells via Activation of miR193a-5p and Inhibition of β-Catenin/c-Myc/Hexokinase 2 Signaling Axis

**DOI:** 10.3390/ijms25179418

**Published:** 2024-08-30

**Authors:** Nari Shin, Hyo-Jung Lee, Deok Yong Sim, Chi-Hoon Ahn, Su-Yeon Park, Wonil Koh, Jaeho Khil, Bum-Sang Shim, Bonglee Kim, Sung-Hoon Kim

**Affiliations:** 1 College of Korean Medicine, Kyung Hee University, Seoul 02447, Republic of Korea; fragendear@naver.com (N.S.); hyonice77@naver.com (H.-J.L.); simdy0821@naver.com (D.Y.S.); ach2565@khu.ac.kr (C.-H.A.); waterlilypark@naver.com (S.-Y.P.); wkoh1231@gmail.com (W.K.); eshimbs@khu.ac.kr (B.-S.S.); bongleekim@khu.ac.kr (B.K.); 2 Institute of Sports Science, Kyung Hee University, Yongin 17104, Republic of Korea; jhkhil@khu.ac.kr

**Keywords:** ginsenoside F2, cervical cancer, apoptosis, glycolysis, miR193a-5p

## Abstract

Though Ginsenoside F2 (GF2), a protopanaxadiol saponin from Panax ginseng, is known to have an anticancer effect, its underlying mechanism still remains unclear. In our model, the anti-glycolytic mechanism of GF2 was investigated in human cervical cancer cells in association with miR193a-5p and the β-catenin/c-Myc/Hexokinase 2 (HK2) signaling axis. Here, GF2 exerted significant cytotoxicity and antiproliferation activity, increased sub-G1, and attenuated the expression of pro-Poly (ADPribose) polymerase (pro-PARP) and pro-cysteine aspartyl-specific protease (procaspase3) in HeLa and SiHa cells. Consistently, GF2 attenuated the expression of Wnt, β-catenin, and c-Myc and their downstream target genes such as HK2, pyruvate kinase isozymes M2 (PKM2), and lactate dehydrogenase A (LDHA), along with a decreased production of glucose and lactate in HeLa and SiHa cells. Moreover, GF2 suppressed β-catenin and c-Myc stability in the presence and absence of cycloheximide in HeLa cells, respectively. Additionally, the depletion of β-catenin reduced the expression of c-Myc and HK2 in HeLa cells, while pyruvate treatment reversed the ability of GF2 to inhibit β-catenin, c-Myc, and PKM2 in GF2-treated HeLa cells. Notably, GF2 upregulated the expression of microRNA139a-5p (miR139a-5p) in HeLa cells. Consistently, the miR139a-5p mimic enhanced the suppression of β-catenin, c-Myc, and HK2, while the miR193a-5p inhibitor reversed the ability of GF2 to attenuate the expression of β-catenin, c-Myc, and HK2 in HeLa cells. Overall, these findings suggest that GF2 induces apoptosis via the activation of miR193a-5p and the inhibition of β-catenin/c-Myc/HK signaling in cervical cancer cells.

## 1. Introduction

Since cervical cancer, a gynecologic malignancy in women [1], has been treated using chemotherapy for years, it has drawbacks including severe toxicity and drug resistance [2]. Hence, with regard to cancer treatment, some natural compounds derived from plants are of note due to selective apoptosis in cervical cancer cells, with less toxicity in normal cells compared to currently used chemotherapy drugs [3].

Previous evidence reveals that cancer cells take advantage of aerobic glycolysis to metabolize glucose [4] in what is called the “Warburg effect”, frequently detected in several cancer cells [5]. Glycolytic enzyme genes such as hexokinase 2 (HK2) and pyruvate kinase isozymes M2 (PKM2) are known to promote cell proliferation due to their high glycolytic metabolism [6]. Also, the phosphoinositide 3-kinase (PI3K)/AKT, Myc, NRF/KEAP1, and Hippo signaling pathways are involved in the cancer metabolism [7,8]. Furthermore, β-catenin/Wnt signaling mediates the glycolysis metabolism in cancer cells [9]. Consistently, β-catenin/Wnt signaling increases aerobic glycolysis by reducing cytochrome c oxidase in breast cancer [10], while the increase in Wnt/β-catenin pathway signaling promotes HK2 and PKM2 in exudative age-related macular degeneration [11]. Similarly, solasonine has been reported to inhibit the glucose metabolism via the suppression of β-catenin/Wnt signaling [12], whereas cryptotanshinone has been shown to reduce glycolysis via PKM2/β-catenin in breast cancer [13].

Generally, ginsenosides from *Panax ginseng* are classified into protopanaxadiol (Rb1, Rb2, Rb3, Rc, Rd, Rg5, Rk1, and Rg3) and protopanaxatriol (Re, Rf, Rk3, Rh4, Rh1, and Rg1) [14]. Though Ginsenoside F2 (GF2) has been shown to exert anti-inflammatory [15], antioxidant [16], anti-obesity [17], and anticancer effects in stomach [18], breast [19], liver [20], and glioblastoma [21] cancers, its underlying apoptotic mechanism has still not been clarified, at least until now. Thus, in our work, the anti-Warburg mechanism of Ginsenoside F2 was elucidated in cervical cancer cells in relation to miR193a-5p and the c-Myc/β-catenin/HK2 signaling axis.

## 2. Results

### 2.1. Cytotoxic and Antiproliferative Effects of GF2 in Human Cervical Cancer Cells

**Figure 1:**
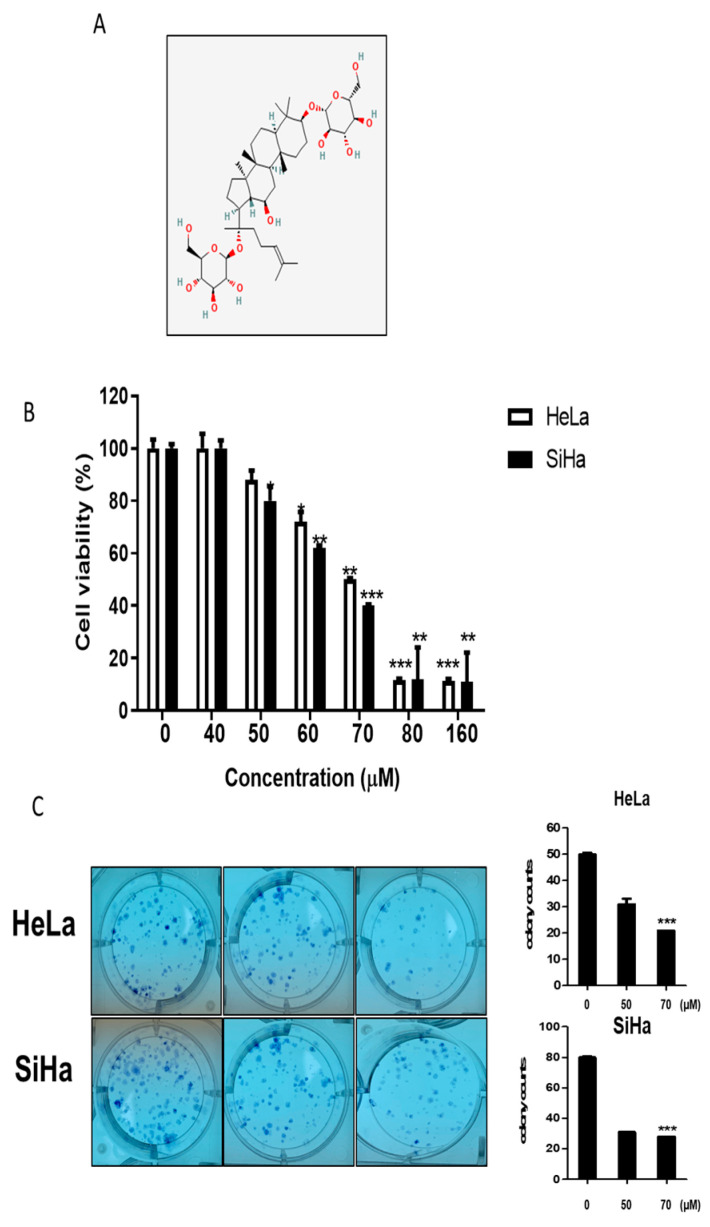
Effect of GF2 on cytotoxicity and colony formation in HeLa and SiHa cells. (**A**) Chemical structure of GF2 (MW = 785.03). (**B**) Cytotoxic effect of GF2 in HeLa and SiHa cells. The cells were exposed to various concentrations of GF2 for 24 h, and cell viability was assessed by MTT assay. * *p* < 0.05, ** *p* < 0.01, and *** *p* < 0.001 versus the untreated control. (**C**) Antiproliferative effect of GF2 in HeLa and SiHa cells. HeLa and SiHa cells were treated with GF2 (0, 50, and 70 μM), and then colony formation took place over 1 week. The colonies were visualized by staining with Diff-Quick solution (Sysmex, Kobe, Japan). The data represent the means ± SD.

To evaluate the cytotoxic effect of GF2 (Figure 1A), a cell viability assay was performed in HeLa and SiHa cells using the MTT assay. The cells were treated with the indicated concentrations of GF2 (0, 40, 50, 60, 70, 80, and 100 μM) for 24 h. GF2 decreased the cell viability in the HeLa and SiHa cells (Figure 1B). Likewise, GF2 significantly decreased the number of colonies in the HeLa and SiHa cells compared to the untreated control (Figure 1C).

### 2.2. GF2 Increased Sub-G1 Population and Induced Apoptosis in HeLa and SiHa Cells

**Figure 2:**
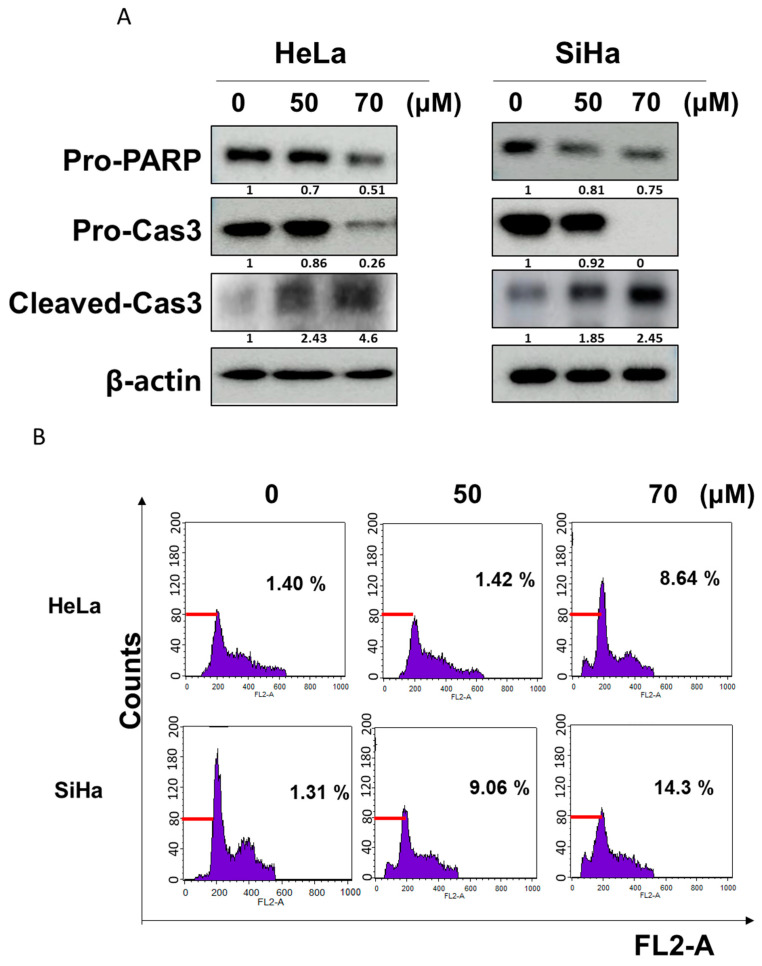
Effect of GF2 on apoptosis in HeLa and SiHa cells. (**A**) Effect of GF2 on PARP and caspase 3 in HeLa and SiHa cells. (**B**) Effect of GF2 on sub-G1 phase in HeLa and SiHa cells.

To confirm the apoptotic effect of GF2, a Western-blot assay and a cell cycle analysis were performed in GF2-treated HeLa and SiHa cells. GF2 decreased the expression of pro-PARP and pro-caspase 3 and increased the expression of cleaved-caspase3 in HeLa and SiHa cells (Figure 2A). As shown in Figure 2B, GF2 increased the sub-G1 population in HeLa and SiHa cells.

### 2.3. GF2 Diminished the Expression Level of Glycolysis Proteins as Well as the Production of Lactate in HeLa and SiHa Cells

**Figure 3:**
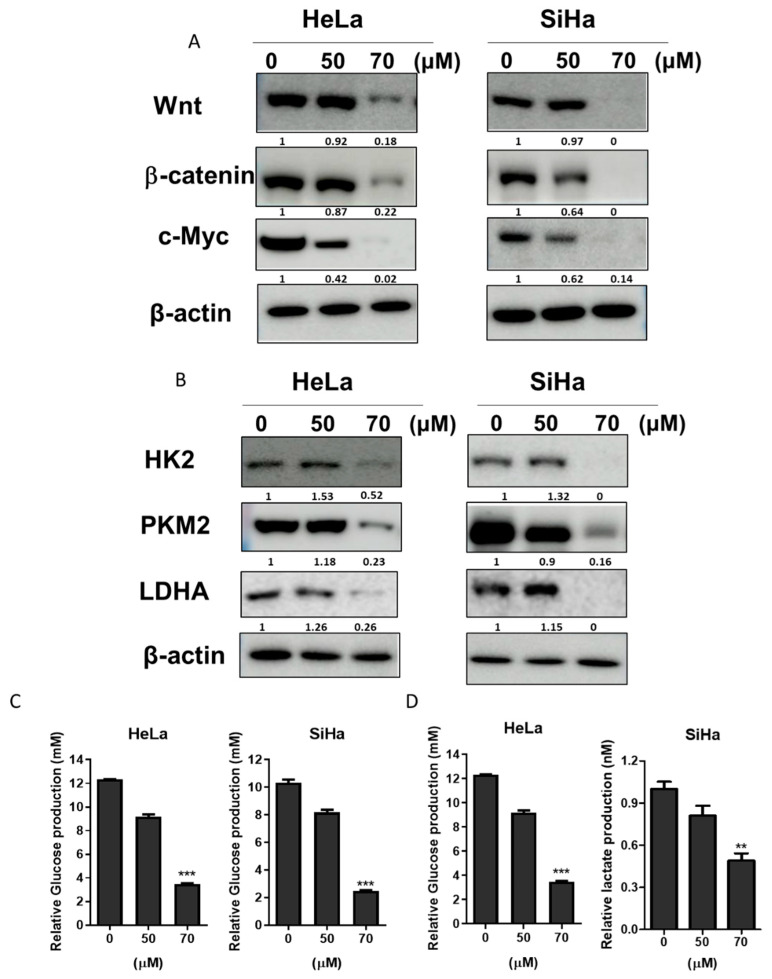
Effect of GF2 on band level of Wnt, β-catenin, c-Myc, HK2, PKM2, and LDHA in HeLa and SiHa cells. (**A**) Effect of GF2 on Wnt, β-catenin, and c-Myc in HeLa and SiHa cells. (**B**) Effect of GF2 on HK2, PKM2, and LDHA in HeLa and SiHa cells. (**C**) Effect of GF2 on glucose production in HeLa and SiHa cells using a colorimetric assay. The results represent the ±SD; *** *p* < 0.001. (**D**) Effect of GF2 on lactate production in HeLa and SiHa cells using a colorimetric assay. The results represent mean ± SD; ** *p* < 0.01, *** *p* < 0.001.

To examine whether the apoptotic effect of GF2 was related to the Wnt/β-catenin/c-Myc pathway or glycolysis signaling, Western blotting was performed in GF2-treated SiHa and HeLa cells. Interestingly, GF2 diminished the band level of Wnt, β-catenin, and c-Myc in SiHa and HeLa cells (Figure 3A). Also, the band level of the PKM2, LDHA, and HK2 glycolytic enzymes was decreased in the GF2-treated HeLa and SiHa cells (Figure 3B). Also, GF2 decreased the levels of glucose and lactate in HeLa and SiHa cells according to the ELISA results (Figure 3C,D).

### 2.4. GF2 Reduced β-Catenin, Glucose-Related Proteins, and c-Myc Stability in HeLa Cells

**Figure 4:**
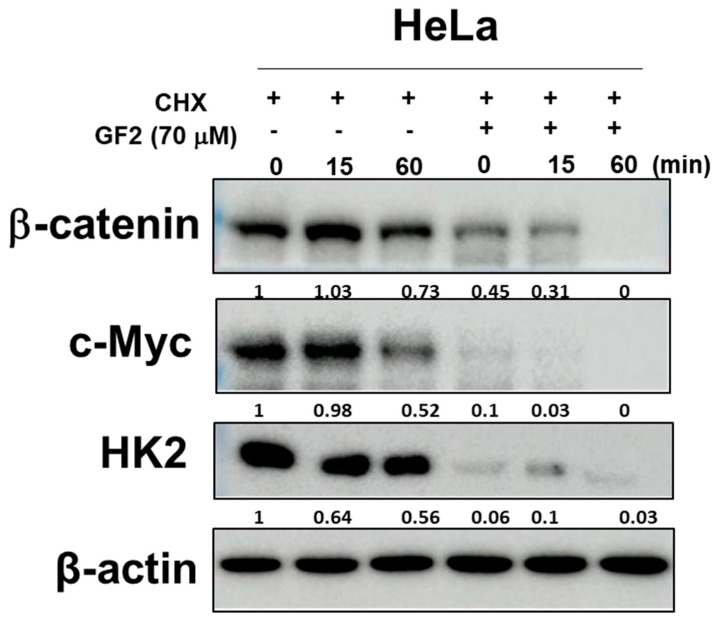
Effect of GF2 on the stability of β-catenin, c-Myc, and HK2 in HeLa cells.

The β-catenin-mediated activation of glycolytic proteins leads to the activation of c-Myc [22], and, consequently, c-Myc upregulates the band level of PKM2, LDHA, and HK2 in cancer cells [23,24]. Here, GF2 diminished β-catenin and c-Myc stability compared to cycloheximide alone, as well as the band level of HK2 in HeLa cells (Figure 4).

### 2.5. GF2 Disrupted the Interaction between β-Catenin and c-Myc, While β-Catenin siRNA Reduced the Band Level of Glycolysis-Associated Proteins and GF2-Induced ATP Depletion in HeLa Cells

**Figure 5:**
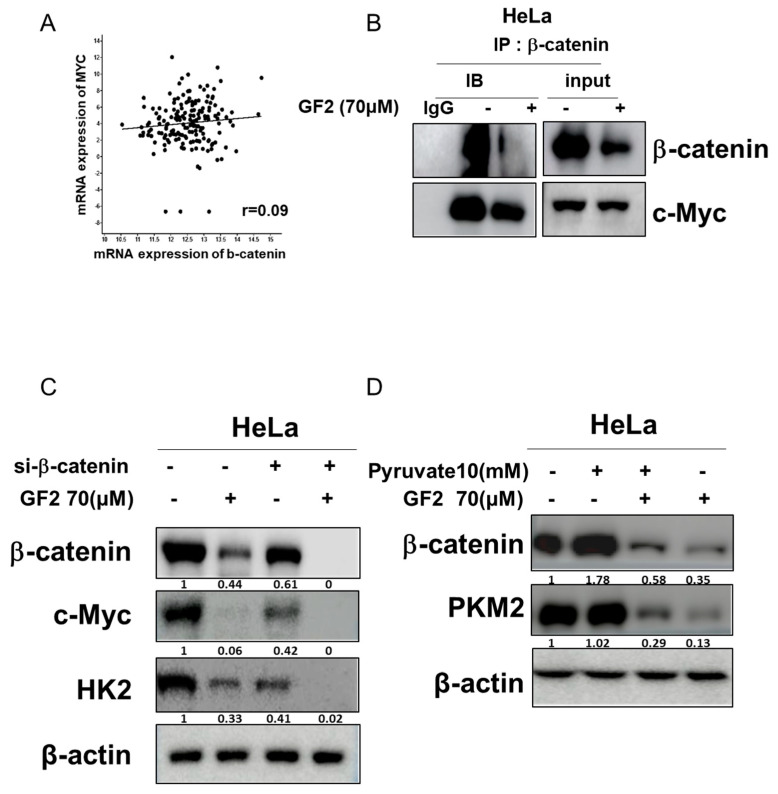
Binding index and correlation efficient between β-catenin and c-Myc and the effect of β-catenin siRNA or pyruvate on glycolysis-related proteins in HeLa cells. (**A**) RNA-seq data (cBioportal) confirm a strong correlation between β-catenin and c-Myc with a Spearman correlation coefficient of 0.09. (**B**) Binding between β-catenin and c-Myc in HeLa cells. Immunoprecipitation was performed in HeLa cells treated with GF2 for 24 h and then subjected to Western blotting to detect β-catenin and c-Myc concentrations in whole-cell lysates. (**C**) Effect of β-catenin depletion on β-catenin, c-Myc, and HK2 in GF2-treated HeLa cells. (**D**) Effect of pyruvate treatment on β-catenin and PKM2 in GF2-treated HeLa cells.

A c-Bioportal database analysis was conducted to demonstrate the correlation between β-catenin and c-Myc, with a Spearman correlation coefficient of 0.09 (Figure 5A). To confirm the interaction between β-catenin and c-Myc, immunoprecipitation was performed in HeLa cells, revealing that, while β-catenin binds to c-Myc in HeLa cells, this was suppressed by GF2 (Figure 5B). Also, to explore the critical role of β-catenin in the glycolysis process, β-catenin siRNA was applied to GF2-treated HeLa cells. β-catenin siRNA diminished the band level of c-Myc and HK2 in HeLa cells (Figure 5A). Previous evidence demonstrates that pyruvate enhances glycolysis, such as TCA cycle flux and ATP production [25,26]. As expected, pyruvate disturbed the ability of GF2 to inhibit PKM2 and β-catenin in HeLa cells (Figure 5B).

### 2.6. miR139a-5p Plays a Critical Role in GF2-Induced Apoptosis in HeLa Cells

**Figure 6:**
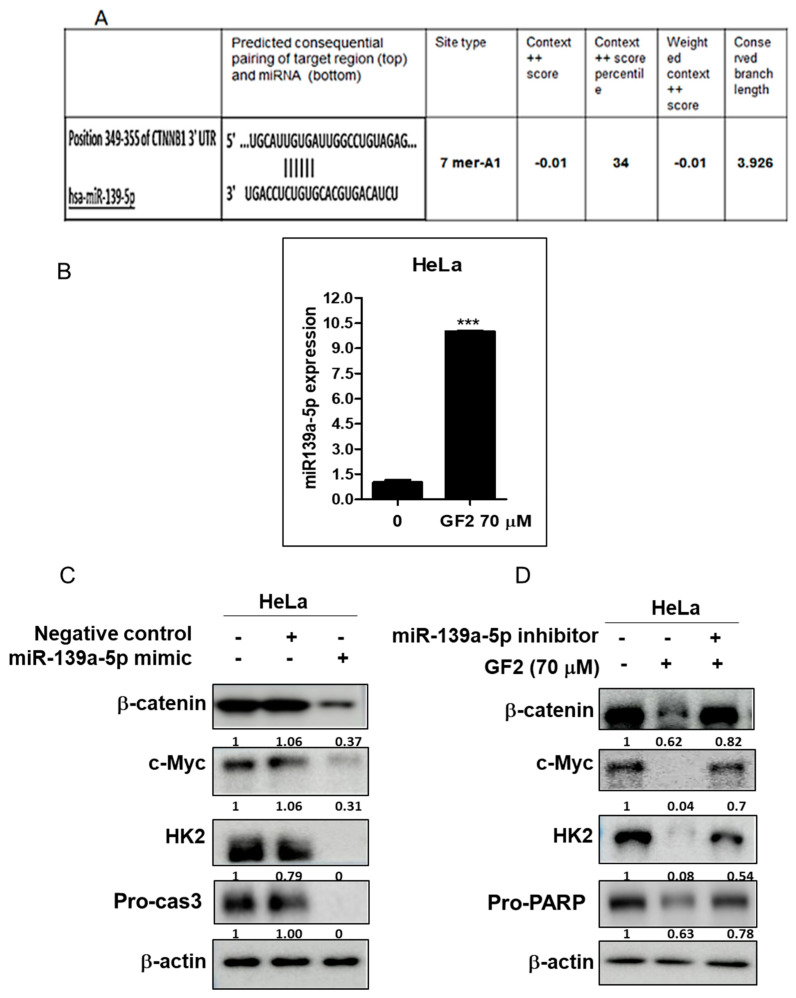
The important role of miR-139a-5p in GF2-induced apoptosis in HeLa cells. (**A**) Direct binding of miR-139a-5p to the 3′-Untranslated region of β-catenin. (**B**) Endogenous expression level of miR139a-5p in HeLa cells. The expression level of miR139a-5p was measured in intact HeLa cells by RT-PCR, *** *p* < 0.001. (**C**) Effect of miR139a-5p mimic on β-catenin, HK2, and c-Myc in HeLa cells. The expression of β-catenin, c-Myc, HK2, and pro-PARP was evaluated in HeLa cells with or without the miR139a-5p mimic by Western blotting. (**D**) Effect of miR139a-5p inhibitor on β-catenin, c-Myc, HK2, and pro-PARP in GF2-treated HeLa cells. The expression of β-catenin, c-Myc, HK2, and pro-PARP was evaluated in HeLa cells with or without GF2 by Western blotting.

To assess the role of miR139a-5p in GF2-induced apoptosis, Western blotting was carried out in HeLa cells. Herein, GF2 incremented the mRNA level of miR139a-5p in HeLa cells. Consistently, the miR139a-5p mimic decreased the band level of β-catenin, c-Myc, and HK2, whereas the miR139a-5p inhibitor reversed the capacity of GF2 to reduce β-catenin, c-Myc, and HK2 in HeLa cells. Indeed, the TargetScan web server for microRNA biological targets revealed that miR-139a-5p directly bound itself to the 3′-Untranslated region of β-catenin (Figure 6A). In addition, GF2 upregulated the mRNA band level of miR139a-5p in HeLa cells (Figure 6B). Furthermore, the miR139a-5p mimic decreased the band level of β-catenin, c-Myc, HK2, and pro-caspase 3 in HeLa cells (Figure 6C), whereas the miR139a-5p inhibitor reversed the capacity of GF2 to reduce the band level of β-catenin, c-Myc, HK2, and pro-PARP in HeLa cells (Figure 6D).

## 3. Discussion

In the present study, the anti-Warburg mechanism of Ginsenoside F2 (GF2), a protopanaxadiol saponin compound derived from Panax ginseng [27], was explored in human cervical cancer cells in relation to glycolysis regulated by miR-139-5p and β-catenin/c-Myc signaling.

Herein, GF2 reduced the viability of HeLa and SiHa cells, indicating the cytotoxicity of GF2 in cervical cancer cells. Also, GF2 significantly decreased the band level of Pro-caspase-3 and Pro-PARP and incremented the sub-G1 phase in HeLa and SiHa cells, implying that its cytotoxicity is exerted by apoptosis in HeLa and SiHa cells.

β-catenin-mediated canonical Wnt signaling is closely involved in cell proliferation, metastasis, and glycolysis [9,10,28], since β-catenin is a key protein of the Wnt signaling pathway [29,30]. The accumulated cytoplasmic and nuclear translocation of β-catenin promotes the proliferation and progression of cancers [31,32]. Furthermore, the activation of Wnt/β-catenin signaling promotes glycolysis with HK2 and PKM2, leading to the Warburg effect [9,33]. Also, β-catenin and c-Myc, as target genes of β-catenin, induce glycolysis in cancer cells [22,34,35]. Interestingly, in this study, GF2 decreased the band level of Wnt, β-catenin, and c-Myc in HeLa and SiHa cells, demonstrating that GF2 transcriptionally inhibits β-catenin/c-Myc signaling during glycolysis in cervical cancer cells. Also, the band level of the HK2, PKM2, and LDHA glycolytic enzymes was decreased in GF2-treated HeLa and SiHa cells, implying that GF2 inhibits glycolytic enzymes, leading to the Warburg effect.

The β-catenin-mediated activation of glycolytic proteins promotes the oncogenic potential of c-Myc [22], since c-Myc upregulates the band level of HK2, LDHA, and PKM2 in cancer cells [23,24]. Here, GF2 diminished β-catenin and c-Myc stability in HeLa cells compared to cycloheximide alone, with a decreased band level of HK2, indicating an inhibitory effect of GF2 on β-catenin/c-Myc/HK2 signaling.

β-catenin/c-Myc signaling is known to promote cancer proliferation, invasion, and the Warburg effect [36]. As expected, β-catenin siRNA reduced the band level of c-Myc, HK2, and pro-caspase3 in HeLa cells, indicating that the suppression of β-catenin inhibits glycolysis and apoptosis in cancer cells.

Pyruvate enhances glycolysis to prevent ATP depletion or cell death [25,26]. Indeed, the pyruvate treatment diminished the efficacy of GF2 in lowering the band level of cMyc, PKM2, and pro-PARP in HeLa cells, implying that the apoptosis induced by GF2 is closely associated with energy depletion in GF2-treated HeLa cells.

miR139a-5p is reported to reduce proliferation and migration via Wnt/β-catenin signaling in cervical cancer cells [37,38]. Herein, GF2 increased the mRNA band level of miR139a-5p in HeLa cells. Also, the miR139a-5p mimic decreased the band level of β-catenin, c-Myc, and HK2, whereas the miR139a-5p inhibitor reversed the capacity of GF2 to reduce β-catenin, c-Myc, HK2, and procaspase 3 expression in HeLa cells, indicating the important role of miR139a-5p in GF2-induced apoptosis and the anti-Warburg effect in HeLa cells.

**Figure 7:**
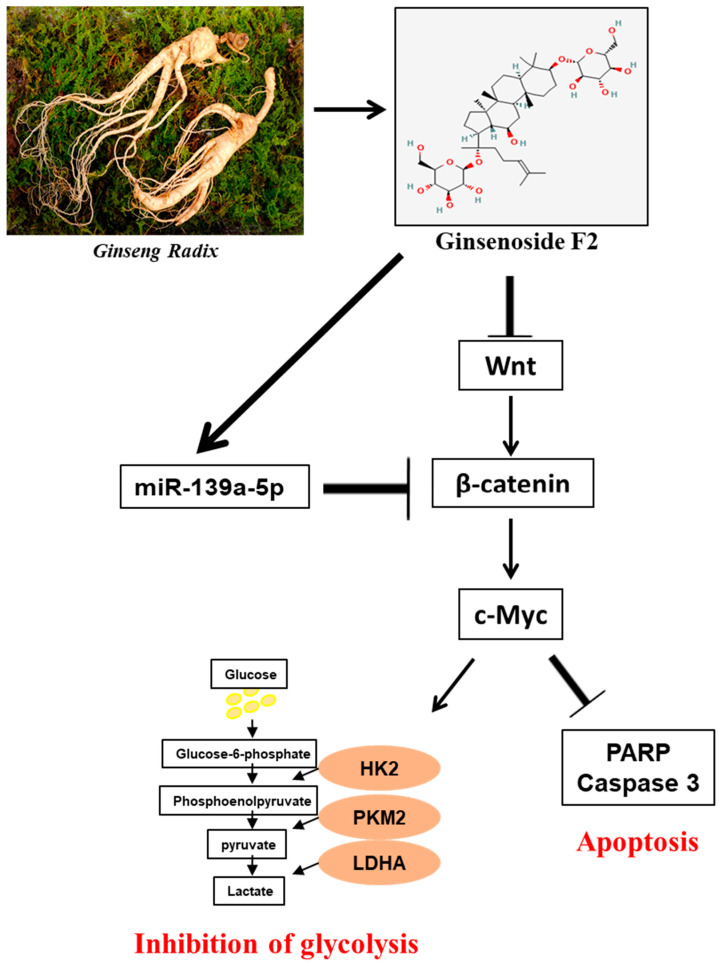
Schematic diagram on the anti-Warburg effect of GF2 via the activation of miR-139a-5p and the suppression of the β-catenin/c-Myc/HK2 signaling axis.

In summary, GF2 increased the cytotoxicity and the sub-G1 phase along with the suppression of pro-PARP, pro-cas3, Wnt, β-catenin, c-Myc, HK2, LDHA, and PKM2 in cervical cancer cells. Also, GF2 attenuated β-catenin and c-Myc stability. Conversely, the pyruvate addition disturbed the apoptotic effect of GF2 in reducing the band level of β-catenin, c-Myc, HK2, and procaspase 3 in HeLa cells. Furthermore, GF2 upregulated miR139a-5p, and the miR139a-5p inhibitor reversed the capacity of GF2 to lower the band level of β-catenin, c-Myc, HK2, and procaspase 3 in HeLa cells. Overall, our results provide evidence that GF2 inhibits the Warburg effect via the upregulation of miR139a-5p and the suppression of the Wnt/β-catenin/c-Myc/HK2 signaling axis, making it a potent chemo-preventative candidate for cervical cancer therapy (Figure 7).

## 4. Materials and Methods

### 4.1. Ginsenoside F2 (GF2)

Ginsenoside F2 (GF2) (Figure 1A; CAS 62025-49-4) was supplied by Santa Cruz Biotechnology. GF2 (stock solution:10 mM) dissolved in 100% dimethyl sulfoxide (DMSO) was kept in a deep freezer (−20 °C) in our lab for subsequent experiments.

### 4.2. Cell Culture

HeLa (ccl-2, ATCC, Manassas, VA, USA) and SiHa (HTB-35, ATCC, Manassas, VA, USA) cervical cancer cells supplied by the ATCC company were cultured in Dulbecco’s modified Eagle medium (DMEM), including 10% FBS and 1% antibiotic antimycotic solution (Welgene, Republic of Korea). These cells were maintained under a humidified atmosphere with 5% CO_2_ at 37 °C.

### 4.3. Cell Viability Assay

To evaluate the cytotoxicity of GF2, HeLa and SiHa cells were seeded onto 96-well tissue culture plates (1 × 10^4^ cells/well) and exposed to various concentrations (0, 40, 50, 60, 70, 80, and 160 µM) of GF2 at 37 °C for 24 h. Subsequently, MTT solution (1 mg/mL) was added to each well for 2 h at 37 °C in the dark and dissolved in 100 μL of dimethyl sulfoxide at room temperature for 10 min. Optical density (OD) was measured using a microplate reader (Molecular Devices, LLC, Sunnyvale, CA, USA) at a wavelength of 570 nm. Cell viability was calculated as a percentage of viable cells in the GF2-treated group, compared to the untreated control.

### 4.4. Cell Cycle Analysis

SiHa and HeLa cells (1 × 10^6^ cells/mL) were treated for 24 h with different concentrations of GF2, as described previously [39]. The cells washed with cold PBS were exposed to RNase A (10 mg/mL), subjected to staining with propidium iodide (PI; 50 μg/mL), and analyzed to determine their cell cycle phases by flow cytometry (BD Biosciences; Becton Dickinson and Company, Franklin Lakes, NJ, USA) using the CellQuest Software (Version 5.2.1.).

### 4.5. RT-qPCR Analysis

As described previously [40], the total RNAs of 1 × 10^7^ cells from GF2-treated HeLa cells were lysed in 1 mL TRIzol, and the cDNA levels were detected using the LightCycler TM instrument with primers including miRNA139-5p-forward (sequence 5′-AGT GCA CGT GTC TCC-3′) and reverse (sequence 5′-GAA CAT GTC TGC GTA TCT C-3′), miR139a-5p inhibitor (sequence (5′-3′):UCUACAGUGCACGUGUCUCCAGU), miR139a-5p mimic (sequence (5′-3′):UCUACAGUGCACGUGUCUCCAGU), and miR Control (Cat.No. SMC-2001) and hGAPDH-plasmids supplied by Bioneer (Daejeon gwangyeoksi, Republic of Korea).

### 4.6. Western Blotting

As described previously [39], HeLa and SiHa cells (1 × 10^6^ cells/mL) treated with different concentrations of GF2 were lysed in a lysis solution (1× protease inhibitor cocktail, 1% Triton X-100, 0.1% SDS, 1 mM EDTA, 1 mM Na_3_VO_4_, 1 mM NaF, 50 mM Tris–HCl, and pH 7.4 150 mM NaCl) on ice. The cell lysates were spun at 14,000× *g* for 20 min at 4 °C. The protein concentrations of the obtained supernatants were measured using the RC DC protein assay kit (Bio-Rad, Hercules, CA, USA). The proteins samples were loaded on 8–15% NuPAGE Bis–Tris gels (Novex, Carlsbad, CA, USA). The membranes were blocked with TBST-diluted 5% skim milk for 1h at room temperature or TBST-diluted 5% BSA for 4 h at 4 °C. Then, these were incubated with primary antibodies of β-actin (Cat No A2228, Merck KGaA, St. Louis, MO, USA), PARP (Cat.No 9542, Cell Signaling Technology, Danvers, MA, USA), Caspase3 (Cat.No 9662, Cell Signaling Technology, Danvers, MA, USA), HK2 (Cat No 2106, Cell Signaling Technology, Danvers, MA, USA), PKM2 (Cat.No 4053, Cell Signaling Technology, Danvers, MA, USA), LDH (Cat No sc-133123, Santacruz, Dallas, Texas, USA), Wnt (Cat.No SC-136163, Santacruz Biotechnology, Dallas, TX, USA), β-catenin (Cat.No SC-7199, Santacruz Biotechnology, Dallas, TX, USA), and c-Myc (Cat No ab32072, Abcam, Waltham, MA, USA) diluted in 5% BSA in TBST overnight at 4 °C, washed three times for 10 min with TBST, and incubated with HRP-conjugated secondary antibodies (Cell Signaling Technology, Danvers, MA, USA) for 2 h. Their expression was visualized using the ECL Immunoblotting detection reagent (GE Healthcare, Little Chalfont, UK).

### 4.7. Co-Immunoprecipitation

HeLa cells were lysed in a lysis buffer (50 mM Tris–HCl, pH 7.4, 0.1% SDS, 150 mM NaCl, 1% Triton X-100, 1 mM NaF, 1 mM EDTA, 1 mM Na_3_VO_4_, and 1× protease inhibitor cocktail) and then immunoprecipitated with β-catenin and the c-Myc antibody. Thereafter, Protein A/G sepharose beads (Santa Cruz Biotechnology, Santa Cruz, CA, USA) were applied. The final precipitated proteins were subjected to immunoblotting with the indicated antibodies.

### 4.8. RNA Interference

HeLa cells were seeded onto a culture plate overnight and transfected with the plasmids of the miR134 mimic, the miR139a-5p inhibitor, and the miR control (200 nM) supplied by the Bioneer company (Daejeon, Republic of Korea) using the Lipofetamine 2000 reagent (Invitrogen, Carlsbad, CA, USA) according to the manufacturer’s protocols. The transfected cells were incubated for 48 h for subsequent experiments.

### 4.9. Statistical Analysis

All the data presented as the means ± SD in the current work were analyzed using GraphPad Prism 8.0 (Dotmatics, Boston, MA, USA). To compare the statistical significance between groups, Student’s *t* test was used. A *p* value of <0.05 between two groups was accepted as statistically significant.

## 5. Conclusions

These findings highlight the apoptotic mechanism of GF2 via its anti-Warburg effect mediated by the activation of miR193a-5p and the inhibition of β-catenin/c-Myc/HK2 signaling in cervical cancer cells.

## Data Availability

All the data and materials supporting the conclusions are included in the main paper.
